# The Effect of Congenital and Postnatal Hypothyroidism on Depression-Like Behaviors in Juvenile Rats

**DOI:** 10.4274/jcrpe.3498

**Published:** 2016-12-01

**Authors:** Erdoğan Özgür, Börte Gürbüz Özgür, Hatice Aksu, Gökhan Cesur

**Affiliations:** 1 Nazilli State Hospital, Clinic of Ear, Nose, and Throat, Aydın, Turkey; 2 Adnan Menderes University Faculty of Medicine, Department of Child and Adolescent Psychiatry, Aydın, Turkey; 3 Adnan Menderes University Faculty of Medicine, Department of Physiology, Aydın, Turkey

**Keywords:** congenital hypothyroidism, depression, forced swimming test, rats

## Abstract

**Objective:**

The aim of this study was to investigate depression-like behaviors of juvenile rats with congenital and postnatal hypothyroidism.

**Methods:**

Twenty-seven newborn rat pups were used. First, 6-month-old Wistar Albino female rats were impregnated. Methimazole (0.025% wt/vol) was given to dam rats from the first day of pregnancy until postnatal 21 days (P21) to generate pups with congenital hypothyroidism (n=8), whereas in the postnatal hypothyroidism group (n=10), methimazole was given from P0 to P21. In the control group (n=9), dam rats were fed ad libitum and normal tap water. Offspring were fed with breast milk from their mothers. The behavioral parameters were measured with the juvenile forced swimming test (JFST). The procedure of JFST consisted of two sessions in two consecutive days: the 15-minute pre-test on day 1 and the 5-minute test on day 2.

**Results:**

Increased immobility and decreased climbing duration were observed in both congenital and postnatal hypothyroidism groups. Decreased swimming duration was detected in the postnatal hypothyroidism group. Both hypothyroidism groups had a lower body weight gain compared with the control group, while the congenital hypothyroidism group had the lowest body weight.

**Conclusion:**

Our results showed that hypothyroidism had negative effects on depression-like behavior as well as on growth and development. Both congenital and postnatal hypothyroidism caused an increase in immobility time in JFST. New studies are required to understand the differing results on depression-like behavior between congenital and postnatal hypothyroidism.

**WHAT IS ALREADY KNOWN ON THIS TOPIC?**

Hypothyroidism has been recognized as an important cause of depression. Adult rats with hypothyroidism have showed increased immobility time in forced swimming test (FST).

**WHAT THIS STUDY ADDS?**

This is the first study that explores behavioral patterns of juvenile rats with congenital or postnatal hypothyroidism in FST.

## INTRODUCTION

The interaction between thyroid hormones and neurobehavioral alterations has been reported in previous studies (1,2). In mammals, this interaction begins in fetal life and continues throughout life. Thyroid hormones play an essential role in the maturation of the central nervous system by increasing trimonoaminergic neurotransmitters and mediating the formation of neuronal branching and synapses (3). A number of studies have highlighted that fetal and neonatal hypothyroidism have a negative effect on the neurodevelopment process (4,5). Retarded locomotor ability as well as hyperactivity were reported as a consequence of experimental hypothyroidism in developing rats (6,7,8). In clinical trials, hypothyroidism and elevated thyroid-stimulating hormone (TSH) levels have been shown to lead to depression in adult patients (9,10,11,12). In a child and adolescent sample, withdrawal, anxiety/depression, mental problems, attention problems and aggressive behavior subscale scores were found to be significantly higher in the congenital hypothyroidism group in which treatment was started at an early age compared with a control group (13). However, the relationship between depression and congenital hypothyroidism is debatable.

The purpose of this study was to investigate the depression-like behaviors of juvenile rats with congenital and postnatal hypothyroidism.

## METHODS

The study has been approved by Adnan Menderes University Animal Experiments Local Ethics Committee for the ethical care and use of animals in research and was conducted on 6-month-old Wistar Albino female rats (210-250 g weight) and their 27 pups provided by Adnan Menderes University Medical Faculty Experimental Animal Laboratory. All animal care and experimental procedures were in accordance with the National Institutes of Health Guide for Care and Use of Laboratory Animals 1985.

The rats were mated with males for fertilization. Vaginal smears were performed for the determination of pregnancy. When semen was detected in the vaginal smears, rats were thought to be pregnant. Pregnant rats were divided into 3 groups.

Group 1 [methimazole (MMI)-induced prenatal hypothyroidism group]: MMI (0.025% wt/vol) was given daily in drinking water to pregnant rats from the first day of pregnancy until postnatal 21 days (E0 to P21) to generate pups with congenital hypothyroidism (n=8). All pups were fed with breast milk from their lactating mothers.

Group 2 (MMI-induced postnatal hypothyroidism group): The pregnant rats were fed ad libitum with water during pregnancy. MMI (0.025% wt/vol) was given daily in drinking water to dam rats from birth. Offspring were fed with breast milk from their mothers to generate postnatal hypothyroidism (n=10).

Group 3 (Control group): The pregnant rats were fed ad libitum and normal tap water without MMI from E0 to P21. Rat pups were fed with breast milk from their lactating mothers (n=9). Pups were kept in the same cage with their own dams until P21.

The observers were blind to the treatment. The last day, pups were weighed.

The rats were placed separately in restricted plastic cages under artificial lighting from fluorescent lamps, with a 12-h light photoperiod and a 12-h dark photoperiod. The room temperature was set at 25 °C constant heat and 45%-55% humidity rates.

MMI (SC-205747A, Santa Cruz Biotechnology, Inc., Dallas, TX) (0.025% wt/vol) was prepared daily and administered via the peroral route in drinking water. This protocol and dosage of MMI administration are typically used for the production of congenitally hypothyroid rats (14,15,16).

Juvenile forced swimming test (JFST) was administered as described by Reed et al (17). On the first day of the experiment, the rats were placed one by one in a tank 40 cm in height, 25 cm in diameter containing water of 23 °C. The animals were left to spend 15 minutes inside the water. The rats were then placed back in their cages and dried. The water in the tank was changed at each animal replacement. Twenty-four hours after this familiarization, the JFST was performed. A high-resolution camcorder (Samsung HMX-QF30 full HD) recorded the 5 minutes following the first minute of contact with the water. The rats were placed back in their cages after they were taken from the tank and dried. The presented procedure was applied to all animals in an identical way. Then climbing, swimming, and immobility durations were determined via the camcorder recordings by a researcher blind to the treatment groups. Modified scoring criteria for juvenile rats were applied (17). 5-minute durations were uniformly divided into 5-second duration intervals and the type of interval was determined according to the dominant activity in the interval (18,19,20). The duration in seconds of each activity exhibited by the experiment animal during the JFST was determined by multiplying the number of the intervals of the corresponding activity type by 5.

JFST was performed to all pups to investigate the depression-like behaviors. The procedure of JFST consisted of two sessions in two consecutive days: the 15-minute pre-test on day 1 and the 5-minute test on day 2 (24 h later). The behavioral parameters analyzed were duration of immobility, swimming, and climbing. After the swimming test was terminated, intracardiac blood samples were collected under anesthesia with 50 mg/kg ketamine and 10 mg/kg xylazine.

Serum concentrations of free 3,5,3’,5’-tetraiodothyronine [free thyroxine (fT_4_)] and free 3,5,3’-triiodothyronine (fT_3_) were measured with electrochemiluminescence immunoassay (ECLIA) by using commercial kits.

### Statistical Analysis

SPSS 20.0 for Windows packaged program was used to analyze the data (21). Suitability for the normal distribution was evaluated by Kolmogorov-Smirnov test. Data were expressed as mean ± standard deviation (SD). The normally-distributed data were analyzed using a one-way analysis of variance (ANOVA). Behavior patterns (swimming and climbing) were analyzed using ANOVA. The Kruskal-Wallis (KW) H test was used as a non-parametric test for immobility duration. A two-tailed p-value <0.05 was considered statistically significant.

## RESULTS

**Figure 1:**
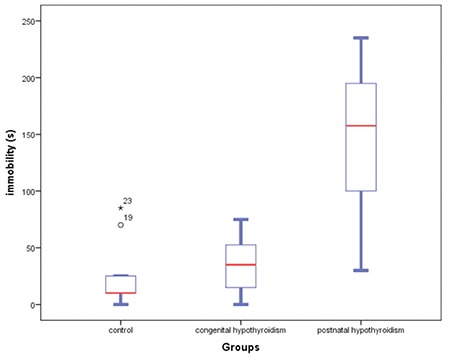
Duration of immobility

In this study, increase in duration of immobility was observed in the postnatal hypothyroidism group compared with control and congenital hypothyroidism groups (KW H test, χ^2^=14.347, df=2, p=0.001; mean ranks: control=8.39, congenital=11.06, postnatal=21.40) (Figure 1).

**Figure 2:**
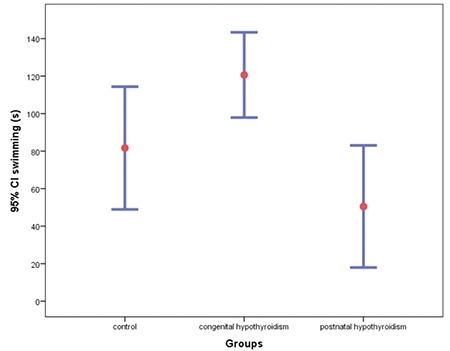
Duration of swimming

There was a statistically significant difference in swimming duration between the groups as determined by one-way ANOVA [F (2.24)=7.438, p=0.003]. A Tukey post-hoc test revealed that swimming duration was significantly lower in the postnatal hypothyroidism group (50.5±45.4 s, p=0.002) compared to the congenital hypothyroidism group (Figure 2).

**Figure 3:**
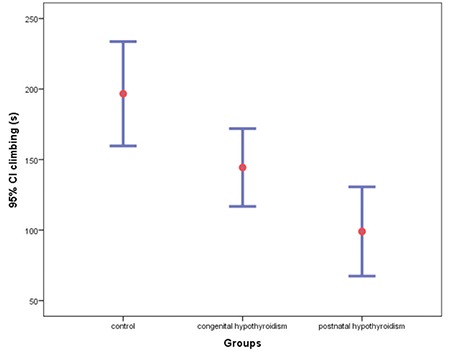
Duration of climbing

In terms of climbing duration, it was lower in the congenital (144.3±32.9 s) and postnatal (99±44.2 s) hypothyroidism groups than in the control group (196.6±48.1 s) (one-way ANOVA [F (2.24)=12.391, p<0.001, Tukey post-hoc test; postnatal x control p<0.001, congenital x control p=0.048]. There was no statistically significant difference between the congenital and postnatal hypothyroidism groups (p=0.085) (Figure 3).

Blood levels of fT_3_ and fT_4_ were lower in the congenital and postnatal groups when compared with the control group, as expected (Table 1). There were statistically significant differences within the groups in weight as an indicator of growth/development (p<0.001) (Table 2).

## DISCUSSION

The forced swimming test is a method that has been accepted and widely used in the assessment of depression-like behavior in rodents (22,23). Prolongation of immobility time in the FST is the main indicator of depression-like behavior (24). In the present study, statistically significantly increased immobility and decreased climbing duration in both congenital and postnatal hypothyroidism groups and decreased swimming duration in the postnatal hypothyroidism group was detected. Consistent with our findings, it has been reported that adult rats with hypothyroidism created using propylthiouracil or hemi/total thyroidectomy, showed increased immobility and decreased climbing time in FST (25,26,27,28,29). Additionally, Ge et al (27) reported reduction in swimming time in both clinical and subclinical hypothyroid rats. In contrast, Yu et al (30) and da Conceicao et al (31) reported decreased immobility time in adult rats with hypothyroidism. As there is a paucity of research evaluating depression-like behavior in juvenile rats with hypothyroidism, we were not able to compare our findings with other studies.

Alterations in both the hypothalamic-pituitary-thyroid and hypothalamic-pituitary-adrenal (HPA) axis have been shown in depression models in rats and in humans (32,33). Stress-induced hyperactivity of the HPA axis, including increased availability of corticotropin-releasing hormone and cortisol may affect the amygdala and hippocampus and may lead to decreased serotonergic neurotransmission (34). Thus, the HPA axis is thought to be the final common pathway in the pathogenesis of depression (35). Montero-Pedrazuela et al (28) suggested that a depressive-like behavior in adult-onset hypothyroidism in rats had a relationship with an impairment of hippocampal proliferation. Interestingly, in our study it was observed that the postnatal hypothyroidism group had the longest immobility time. Johnson et al (36) found that duration of hypothyroidism had different effects on the HPA axis in rats. According to their study, short-term hypothyroidism was associated with increased pituitary corticotroph responsiveness to corticotropin-releasing hormone in contrast with long-term hypothyroidism (36). We speculate that when an imbalance of thyroid hormone homeostasis is acquired, duration of exposure to hypothyroidism would be a possible reason for the different findings in behavioral tests.

There are many animal studies suggesting that thyroid hormones influence norepinephrine and serotonin levels which play crucial role in depression pathogenesis (31,37,38,39,40). A recent study, which aimed to explore the underlying mechanism of a link between thyroid and serotoninergic system, suggested that the lateral habenula might play a role in depression-like behavior in rats with hypothyroidism (29). Hassan et al (41) observed that there was a significant decrease of plasma dopamine, norepinephrine, and serotonin levels in young and adult rats with hypothyroidism. A similar decrease in platelet serotonin concentration was also reported in a study conducted on hypothyroid patients (42). Congenital hypothyroidism leads to a lower developmental quotient and delay in psychomotor development as well as to high depression/anxiety scores in clinical samples (13,43,44,45).

Determination of lower fT_3_ and fT_4_ blood levels in the congenital and postnatal hypothyroidism groups compared with the control group provided an evidence of hypothyroidism. This finding is expected to be accompanied by an increase in TSH levels. Our failure to analyze the rat-specific TSH is the most important limitation of this study.

Thyroid hormones play an important role in growth, development, and neurodevelopmental processes. It has been reported that brain and bone growth and sexual maturation are more affected in rats with thyroid hormone deficiency which has an onset in fetal life (4). Both hypothyroidism groups had a lower body weight gain compared with the control group, while the congenital hypothyroidism group had the lowest body weight. It is thought that growth and development are more affected due to earlier onset of thyroid hormone deficiency in congenital hypothyroidism.

In conclusion, to our knowledge, this is the first study that explores behavioral patterns of juvenile rats with congenital or postnatal hypothyroidism in JFST. Our results showed that hypothyroidism had negative effects on depression-like behavior as well as on growth and development. In both congenital and postnatal hypothyroidism groups, increased immobility time in JFST was observed. We found that in juvenile rats, postnatal hypothyroidism was more likely to cause a depression-like behavior, while congenital hypothyroidism affects mainly the growth and development processes. New studies are required in order to understand the differing results in depression-like behavior between subjects with congenital and postnatal hypothyroidism.

## Acknowledgment

The authors would like to thank Mustafa Yılmaz, MD for biochemical analysis and Serdar Aktaş (veterinarian) for technical support during the experiment.

## Ethics

Ethics Committee Approval: Adnan Menderes University Animal Experiments Local Ethics Committee, 21.04.2014 64583101/2014/047.

Peer-review: Externally peer-reviewed.

## Figures and Tables

**Table 1:**
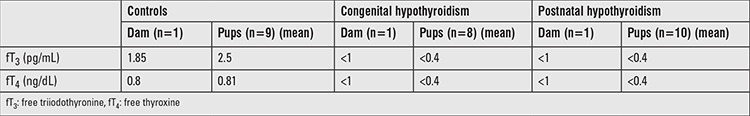
Blood levels of free triiodothyronine and free thyroxine in the 3 groups

**Table 2:**
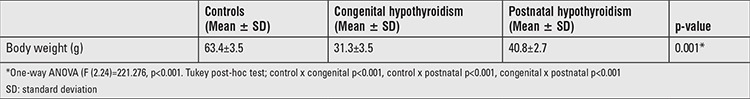
Body weight of the animals in the 3 groups
